# Stiffness‐Independent Toughening of Beams through Coaxial Interfaces

**DOI:** 10.1002/advs.201800728

**Published:** 2018-10-07

**Authors:** Jochen Mueller, Jordan R. Raney, Dennis M. Kochmann, Kristina Shea

**Affiliations:** ^1^ Engineering Design and Computing Laboratory ETH Zurich 8092 Zurich Switzerland; ^2^ Architected Materials Laboratory University of Pennsylvania Philadelphia PA 19104 USA; ^3^ Mechanics and Materials Group ETH Zurich 8092 Zurich Switzerland; ^4^ Kochmann Research Group California Institute of Technology Pasadena CA 91107 USA

**Keywords:** architected materials, cellular structures, core–shell structures, energy absorption, fracture mechanics

## Abstract

To be of engineering relevance, it is essential for stiff and strong materials to possess also high toughness. However, as these properties are typically mutually exclusive, they are rarely found in nature and synthetic replications are extremely limited. Here, an elegant albeit simple physical principle that enables ligaments in cellular networks to possess these mechanical properties simultaneously is presented. The underlying architecture consists of multiple, coaxially aligned layers separated by interfaces that prevent crack propagation, hence increasing the energy required for complete rupture. The results show that the fracture strain and toughness can be increased by over 100%, when compared to conventional reference struts, while fully maintaining the density, stiffness, and strength. The bioinspired and highly versatile approach is scale‐independent under the absence of shear, applicable to various geometries, and complementary to existing approaches. It can, therefore, significantly improve safety and reduce cost and environmental impact in numerous applications, such as packaging, sports equipment, and transportation.

High stiffness, strength, and toughness in combination with low density are properties that are typically mutually exclusive, but often required in a wide range of engineering applications.^1^, ^2^, ^3^ Nature finds ways to combine these properties by growing complex, multiscale architectures that are challenging to replicate synthetically.^4^, ^5^ On the material level, a higher energy absorption can be reached with minimal sacrifice in stiffness by incorporating fillers into a matrix material to impact phenomena like crack bridging or delamination.^3^, ^6^, ^7^, ^8^ More specialized processes, such as freeze casting, can reach even better property combinations through a well‐controlled dispersion or higher filler ratios, which, for example, increase the crack length, hence the energy requirement, through deflection.^4^, ^9^, ^10^ To improve the properties to beyond what is possible by material composition alone, geometric features are added by manipulating the spatial distribution. For example, removing material where it is not required or not as efficiently utilized as in other places increases the efficiency relative to the weight. This can also be done in bulk by spatially tessellating unit cells to create metamaterials with a periodic (micro‐)structure, macroscopically behaving like a homogeneous material with tailorable effective properties. In case of cellular structures, those effective properties are typically defined by their cellular architecture and the properties of the solid constituents.^11^ With relation to energy absorption, cellular structures have been extensively covered both theoretically and experimentally.^12^, ^13^, ^14^, ^15^, ^16^, ^17^, ^18^ Historically, stochastic foams were the dominant type used for energy‐absorbing applications.^19^, ^20^ With the advent of advanced manufacturing methods, researchers started to take advantage of defined and often discrete architectures that can be tuned to specific requirements.^21^, ^22^, ^23^ Herein, the mechanical behavior of a lattice structure is typically described by the unit cell type: stretching‐dominated cells with a high connectivity support stiffness and bending dominated cells with typically a low connectivity are used for energy absorption.^22^, ^24^, ^25^ The performance of each cell can be further improved by optimizing strut geometry and base material composition.^26^, ^27^ With the aim of increasing the stiffness for a given unit cell relative to its weight, a typical approach is to cover the struts with a stiff material while keeping their cores hollow.^11^, ^14^, ^15^, ^28^, ^29^, ^30^, ^31^ When it comes to energy absorption, the material selection has traditionally been the main tuning parameter.^22^ Efforts have been made on the processing side to create solutions to enable the processing of ductile materials such as aluminum,^12^, ^21^ and on the materials side to create tougher materials, for example through particle‐reinforcement.^6^, ^7^, ^32^, ^33^, ^34^ More recently, lightweight cellular architectures have been explored for energy absorption from nano to macroscales^17^, ^18^, ^35^, ^36^, ^37^, ^38^ as well as for improved fracture toughness.^39^, ^40^, ^41^ In particular, additive manufacturing is being exploited to, e.g., coaxially extrude materials into struts and woodpile structures with stiff shells, ductile cores, and unbonded interfaces to promote energy absorption.^18^ Those approaches usually utilize optimized truss topology and architecture as well as small‐scale size effects.^18^, ^35^, ^36^, ^37^, ^39^, ^40^, ^41^

In this work, we take a new investigation route and add geometric features at the individual strut level, without changing the topology or material composition of the structure, by splitting the struts into multiple, coaxially aligned layers (**Figure** **1**a). By preventing bonding between layers, each layer in the strut fails individually when its respective maximum strain is reached. This leads to more benign failure of the strut over a range of strains rather than catastrophic failure when the maximum strain in the outermost layer is reached (Figure 1b). Allowing the strut to fail over a range of strains greatly enhances the toughness of the strut. Despite the radial separation between layers, assuming the gap between layers remains negligible compared to the strut diameter and no significant slippage, the sum of the second moments of area and, hence, the stiffness of the strut, remains unaffected when compared to conventional, monolithic struts. As some fabrication methods require a layer thickness greater than zero, we also discuss the changes in energy absorption and stiffness when the distance between layers is allowed to increase, enabling the designer to find the best trade‐off.

**Figure 1:**
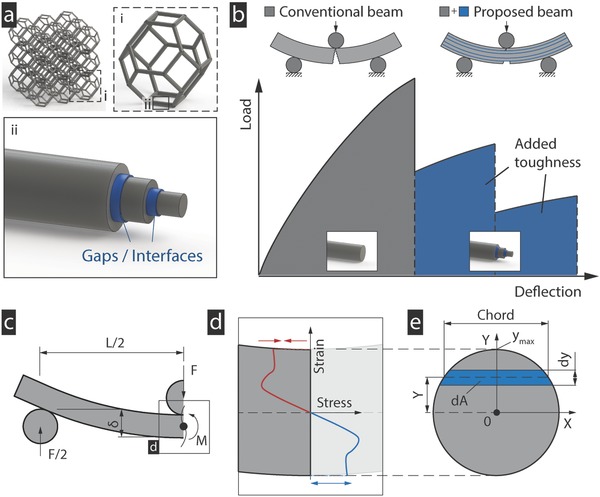
Principle and model. a) Lattice structures consist of multiple hierarchy levels, each of which affects the global response differently. The proposed beams or struts are composed of a model material (gray), separated into one or more coaxially aligned layers isolated by intersections (blue). When compared to conventional beams, the added gaps in the new design allow each layer to fail when its respective failure strain is reached, rather when the failure strain in the outermost layer is reached leading to catastrophic failure. b) Hence, the total failure strain is increased, which results in an increase in absorbed energy when fracturing the structure. c) A strut under bending, from which the global moment, *M*, is calculated. d,e) The model can read the full stress–strain curves of the material as inputs to calculate the resulting load–displacement curve of the beam.

We present an analytical model that computes the complete load–displacement response of rods and multilayered struts under bending. Rather than making simplifying assumptions about the constitutive behavior of the base material, like linear elastic or elastic‐perfectly‐plastic, as is typical,^42^ the model is based on the full, experimentally determined stress–strain curve. To this end, our model takes into account the deformation of the strut by integrating over the complete deflection history. Since the computational cost increases exponentially with the number of layers, we apply mathematical optimization and present a detailed study on the role of the positions, numbers, and thicknesses of the gaps for different classes of engineering materials. The model is validated through bending experiments on polymethyl methacrylate (PMMA) beams of different configurations.

The analytical model relates the local stresses in the cross‐section to the global bending moment, *M*, as calculated from the free body diagram, where *L* is the length of the beam, and calculates the force, *F*, for the given deflection (Figure 1c)^42^

(1) $$M\;=\;-\frac{FL}{4}$$

As opposed to, e.g., the linear elastic assumption, the stresses in our model are not assumed to increase linearly away from the neutral axis to the bottom and top of the cross‐section. Instead, we directly use the nonlinear stress–strain relation of the base material, which can also distinguish between any tension/compression asymmetry on the two sides of the neutral axis (Figure 1d). By integrating the forces, d*f*, across the cross‐sectional area of the beam with their respective distances from the neutral axis, *y*, the moment is calculated. Since d*f* is equal to the product of the stress, σ_(*y*)_, and area, d*A*, the stress distribution results in the total moment

(2) $$M=\int_{A}ydF=\int_{A}y\sigma_{\left(y\right)}\;dA$$

Due to the symmetry of the considered struts and the loading, the neutral axis is always in the center. As σ_(*y*)_ is equal for every *y*, d*A* must only be calculated for each chord of the cross‐sectional circle. Describing the circular cross‐section as a function of the in‐plane coordinates and a constant radius, *x* ^2^ + *y* ^2^ = *r* ^2^, the chord length, *c*_(*y*)_, can be expressed as a function of *y* (Figure 1e)

(3) $$c_{\left(y\right)}=\;2\sqrt{r^{2}-y^{2}}$$

Integrating the chord length over the height, as defined by two coordinates normal to the neutral axis, *y*_1_ and *y*_2_, d*A* is calculated as

(4) $$dA=\int_{y_{1}}^{y_{2}}c_{\left(y\right)}\mathrm{dy}$$

In a similar manner, the area of the shells can be calculated by subtracting the chord of the inner circle from that of the outer circle. Combining Equations (2) and (4), the moment is calculated as

(5) $$M=\int_{-y_{\max}}^{y_{\max}}y\sigma_{\left(y\right)}c_{\left(y\right)}dy$$

Replacing *M* with Equation (1) yields the flexural load–displacement curve as a function of the given stress–strain curve of the material

(6) $$F\;=\frac{4}{L}\;\int_{-y_{\max}}^{y_{\max}}y\sigma_{\left(y\right)}c_{\left(y\right)}dy$$

The energy required to deform the strut, *U*, is represented by the area under the load–displacement curve, which is integrated over the deflection as

(7) $$U\;=\frac{4}{L}\int_{0}^{\delta_{\max}}\int_{-y_{\max}}^{y_{\max}}y\sigma_{\left(y,\delta \right)}c_{\left(y\right)}dyd\delta$$

The overall modulus of the strut is calculated as

(8) $$E_{\mathrm{tot}}=\frac{I_{1}E_{1}+I_{2}E_{2}+\ldots +I_{n}E_{n}}{I_{tot}}$$

with the total second moment of area, *I*_tot_, of a circle being

(9) $$I_{\mathrm{tot}}\;=\frac{\pi }{4}\;{\left(\frac{d}{2}\right)}^{4}$$

The objective, *U*(*r_i_*), of the optimization is to maximize the energy required (Equation (7)) when bending the strut by finding the optimized number of layers and their respective positions in the cross‐section, described by the outer radius, *r_i_*, of the layers, *i*. The objective is subject to constraints given by the thickness of the interfacial layers, *t*_gap_, and the minimum layer thickness, *t*_min_, as dictated by the fabrication process, and the maximum radius, *r_n_*. When there are multiple layers, the radius of each subsequent layer must not be smaller than its own radius (inequality constraint).

Variables:

$$r_{i}={\left(r_{1},\;r_{2},\;\ldots ,\;r_{n}\right)}^{T}\;\mathrm{where}\;i\in {\mathbb{N}}_{0}$$

Objective:

max*U*(*r_i_*) (from Equation (7))

Subject to:

Boundary conditions

$$t_{\min}\leq r_{i}\leq r_{n}$$

Inequality constraints:

$$r_{i-1}+t_{\mathrm{gap}}\leq r_{i}$$

Since the input stress–strain curve is given in the form of discrete, numerical data, which we do not interpolate for reasons of accuracy, a large number of local maxima exist, limiting the choices of appropriate optimization algorithms. Further selection criteria are the constraint type (bound and inequality constraints) and variable type (discrete). A comparison of methods that both satisfy the criteria and are readily implemented resulted in Pattern Search^43^ offering the best trade‐off between accuracy and computational cost, which is therefore used throughout this work. The optimization results are verified against the manually generated solutions for the cases of one and two variables and are >99.9% accurate.

For the layered struts, the experimental results show a rapid crack propagation in the material that is stopped by the intersections, leading to the expected, layer wise failure (**Figure** **2**a). Comparing the mechanical response of the two‐layer rods to that of a conventional rod (i.e., a reference rod with the same cross‐section, but made out of a monolithic layer without gaps), the load–displacement curves are comparable up to the initial fracture, with identical moduli and strength values (Figure 2b,c). Past that point, the conventional rod fails completely, whereas the core of the layered rod continues to sustain loads until its own fracture strain is reached. In the case of the 0.5 core‐to‐shell ratio, the load drop is relatively large, but the total fracture strain is increased by 95%, increasing the energy for complete fracture (measured as the area under the curve) by 20% (Figure 2b). For the larger ratio, the load drop is smaller while the fracture strain is increased by 60%. The energy required for fracture in this case is increased by over 50% (Figure 2c). The three‐layer strut fails in three steps and has a total increase in failure strain and energy absorption of 130% and 60%, respectively (Figure 2d). All cases show good agreement with the numerical results, which, in some cases, tend to underestimate the increase in the reachable energy absorption and fracture strain in a conservative manner.

**Figure 2:**
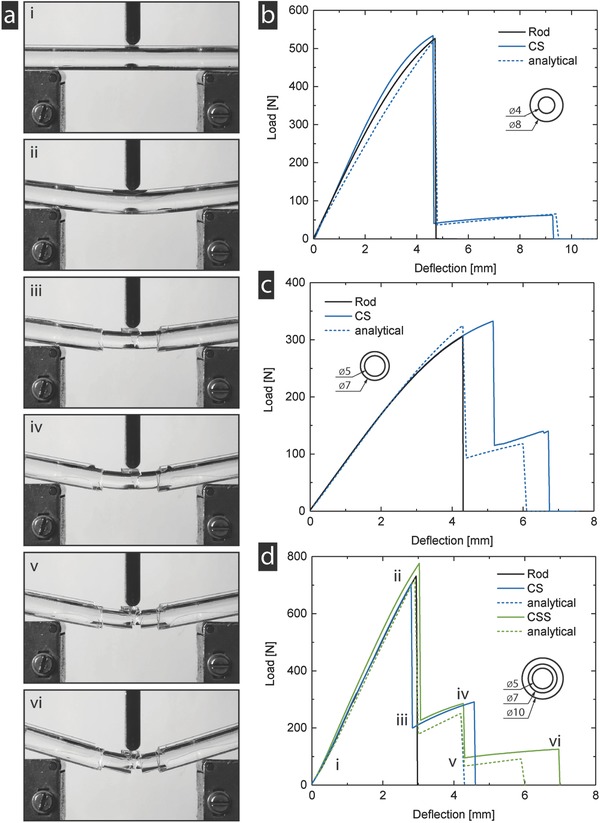
Experimental verification (PMMA). a) Compared to conventional rods of the same material, flexural tests of the presented design show a consecutive failure of the layers. The outer diameter of the rod is 10 mm. b) For a small core‐to‐shell ratio, the total fracture strain is almost doubled, while the drop from the peak load is relatively high. c) For a larger ratio, the added failure strain is smaller, but the drop from the peak stress is smaller. While each case has its own advantages, both of them show a significant increase in the energy required to fracture the rod, with no sacrifice in strength and stiffness. d) Adding a third layer combines the advantages of the two‐layer cases while further increasing the energy absorption. In all cases, the analytical model shows good agreement with the experimental results, with a tendency to underestimate the gained energy absorption and failure strain.

The numerical model allows further exploration of the design space by computing the results for all possible core‐to‐shell ratios in discrete steps (**Figure** **3**a,b). In the case of one intersection with no separation thickness, an increase in energy absorption relative to the conventional strut is observed at ratios starting from 0.2 (Figure 3a). The relative increase grows until a maximum of 25% is reached at a ratio of 0.71 (Figure 3a), before it drops to the value of the conventional strut at a ratio of 1. The ratio of 0.71, computed with our model that can read arbitrary stress–strain curves, is slightly below the optimal ratio of 0.75 for perfectly linear‐elastic material systems, as derived analytically in the Appendix.

**Figure 3:**
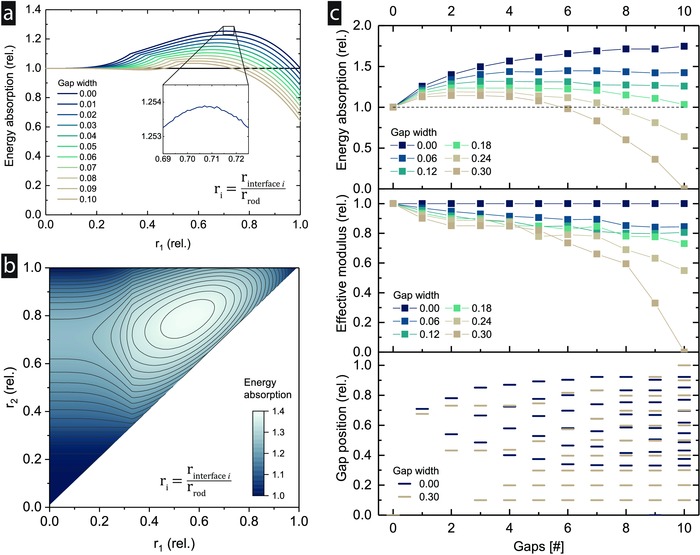
Optimization results (PMMA). a) Sweeping the design space for the tested PMMA material with one intersection shows a possible increase in energy absorption of 25%, depending on the thickness of the gap. b) The relative radii, *r_i_*, are the ratio of the interface radius and the outer radius of the rod. In case of two gaps of zero width, the possible increase in energy absorption reaches 40%. For three or more gaps, the computational cost quickly increases, making manual generation of all options infeasible. c) The optimization results show that thinner gaps are advantageous, and that more layers are generally better. An optimum exists for each gap width larger than zero. The results also show that small gaps reduce the modulus less than larger gaps, which eventually remove all the available material, yielding a modulus of zero. The optimized gap diameters (c, bottom) indicate saturation with an increasing number of gaps, as more of the lower and ineffective diameters are populated.

With the model, we also investigate the effect of a finite separation thickness, which could be the case in fabrication methods other than the one used in this work, such as additive manufacturing. For this case, we assume the worst‐case scenario, in which the gap is not filled with a structural (load‐bearing) material. As hypothesized, the maximum possible increase drops with increasing gap size, but can still reach a significant increase even at gap sizes of 0.1 of the strut diameter (Figure 3a). With increasing gap size, the optimal core‐to‐shell diameter ratio decreases to values smaller than 0.71. When a gap is added at larger core‐to‐shell ratios, the relative energy absorption drops below 1. This is due to pure material removal on the outside of the strut instead of creating a layered system.

In the two‐variable sweep with two gaps, the design space is increased, and the additional variable allows one to further increase the maximum achievable energy absorption (Figure 3b). As before, if the gap width of both layers is zero and the ratio set to 0 or 1, the energy is not increased. This is also true for more layers, meaning that additional layers can only improve the results. The maximum for the two‐variable system is reached at relative ratios of *r*_1_ = 0.58 and *r*_2_ = 0.8 for the first and second layer, respectively. As before, these ratios are slightly smaller than the optimal ratios for a perfectly linear‐elastic material, where they equal 0.62 and 0.83, respectively (see the Appendix). As both of these ratios differ from the one‐variable case, it becomes clear that interaction effects exist. This means that an optimized performance cannot be reached by simply adding more layers, but only by recalculating the positions of all layers, including the existing ones.

While a variable sweep is possible for one or two variables, the exponentially increasing computational cost makes it infeasible to calculate it for more variables. As the sweep is always an approximation based on discretely spaced points on the curve, the optimization results can also be more accurate as it is able to find maxima at any point on the curve. For these reasons, the maximum possible increases were computed with an optimization model for 0 to 10 gaps (Figure 3c). As hypothesized, the maximum achievable energy increases further for the case of zero gap width, eventually plateauing at an increase of 75%. The rise in energy absorption is relatively steep at the beginning and becomes smaller toward the end of the curve, due to the reduced design space available. For gap sizes larger than zero, an optimum must exist, as, at some point, the whole strut will consist of gaps only. The larger the gap width, the fewer layers are required to reach the maximum, and the smaller the maximum increase. In terms of the effective modulus, no reduction is seen for zero gap width. For any gap width larger than zero, the modulus and hence the stiffness of the strut become smaller. For small thicknesses, the increase in energy absorption outweighs the decrease in the modulus, which is the opposite case for larger gap widths. The gap positions indicate the (relative) diameters optimized for maximum energy absorption for each number of possible layers. We see that, for larger gap widths, e.g., 0.30, and increasing numbers of gaps, small ratios between 0 and 0.2 are populated (Figure 3c, bottom). This range has shown to be ineffective in terms of increasing energy absorption (Figure 3a) and can be used for unneeded layers, which would otherwise have a negative effect. Eventually, this leads to a hollow strut where only a thin shell remains, the mechanism typically seen to maximize relative stiffness and strength in lattices.^11^, ^17^, ^44^

It is also important to note that the above mechanism is scale‐independent within the bounds of the Euler–Bernoulli beam theory, i.e., the architectural design principle and resulting crack arrest mechanism do not emerge from size‐dependent phenomena recently explored for achieving high strength or toughness in nanoscale lattices.^17^, ^45^

Next, different families of technologically relevant materials are investigated to draw more general conclusions about the architectural principle proposed. Specifically, samples from brittle, tough, and flexible material groups are selected, as these groups represent most materials found in engineering applications. Brittle and flexible materials tend to have little to no plastic deformation before (catastrophically) failing, with the difference that brittle materials are stiff and strong with little failure strain and flexible materials have the opposite characteristics. Ductile materials, on the other hand, typically exhibit significant plastic deformation, often linked with benign failure. Setting the requirements to a span length between the supports, diameter, and deflection that yield a maximum strain of 15%, common engineering materials with failure strains ranging from well below to above 15% are selected (**Figure** **4**a). The optimization results for zero gap width show that the fiber‐reinforced epoxy (FR epoxy), which is the most brittle material from our selection, reaches the highest increase in relative energy absorption, approaching 70% (Figure 4b). No increase is seen for thermoplastic polyurethane (TPU), and intermediate values are reached for all other materials, suggesting that the relationship depends on the materials' failure strain. Indeed, if we look at the strain distribution, the design space to distribute the additional layers is zero for the TPU case, as no fracture occurs. For the other cases, the core‐to‐shell ratio above which the material's failure strain is reached decreases with decreasing failure strain. This relationship can be further explored by fixing the material and varying the maximum required strains (Figure 4c–e). As the strains are calculated by the span length, strut diameter, and deflection, the presented values represent realistic ranges for different combinations of the properties. The brittle material, represented by the FR epoxy, does not show an interaction effect between the strain and the gaps, meaning that a further increase or decrease in strain will not affect the maximum reachable energy absorption (Figure 4c). For the tough material, represented by the Polyamide 12 (PA12), an interaction effect can be seen at values higher than 15% strain, which equals the failure strain of the material. Below that value, the effect is similar to the previously discussed case of TPU (Figure 4d). Above 15% and below 42%, a transition region is observed where both an increase in gaps and strain yields a higher energy requirement. This is given due to the downward shift of the core‐to‐shell ratio, below which no fracture occurs due to the material's failure strain. In the case of the flexible material, represented by the TPU, the trend shifts further up, displaying a transition zone starting at 30% and going up to >48% (Figure 4e). As the tested strain of 15% is below the transition, no improvement is seen.

**Figure 4:**
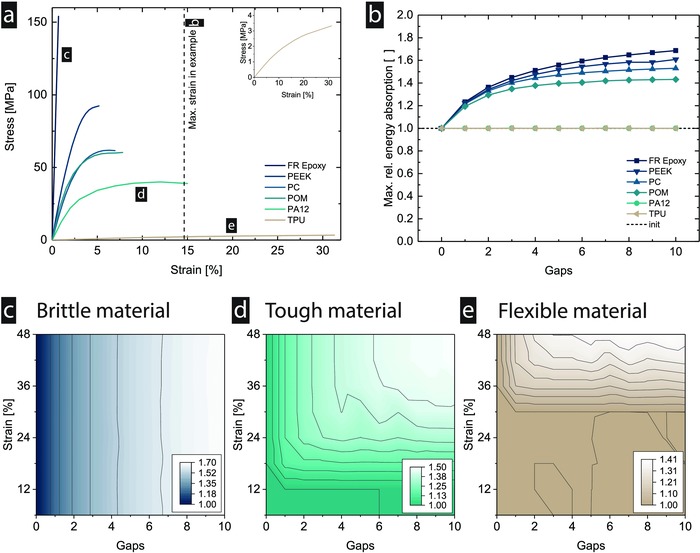
Different classes of engineering materials. a) The model is implemented for a selection of typical engineering materials. b) The results show that the maximum increase in energy absorption depends on the modulus, strength, and failure strain, which needs to be smaller than the bending strain reached in the strut. c) Brittle materials show an improvement at relatively small bending strains. d) Tough materials have a clear transition zone. e) The transition zone is shifted upwards for flexible materials. The brittle materials are represented by the FR epoxy, the tough materials by the PA12, and the flexible materials by TPU. The base material data are retrieved from ref. 46.

The principles developed here also hold if one replaces the gaps with separation layers comprised of soft or even active materials, as long as they do not promote the propagation of cracks across layers.^18^ This would allow the design of multifunctional lattices while maximizing mechanical properties such as energy absorption and stiffness.

Through a relatively simple yet powerful architectural design principle, we have shown how struts in cellular networks can be equipped with significantly higher fracture toughness without sacrificing stiffness and strength. Crack arrest mechanisms, commonly exploited in composite materials, are introduced at the individual strut level resulting in a stepwise strut failure under loading that can also serve as an easy‐to‐detect, early‐warning mechanism signaling the risk of complete failure. The shown example of circular beams serves as an example that can now be generalized and extended in various directions. The principle of introducing geometric interfaces is scale‐independent, under the condition that shear is negligible, and can be applied to other structures and geometries. Applications are expected in numerous areas of engineering, such as transportation, packaging, and sports equipment, and include car bumpers, cushioning in packaging, and sports helmets. Specifically, the increased performance at no additional mechanical cost can significantly improve the safety and efficiency of architected materials and cellular structures.

## Experimental Section

To verify the accuracy of the model, three‐point bending tests are conducted on individual PMMA (PMMA XT, Amsler & Frey AG, Switzerland) struts. PMMA has an elastic modulus of 3.3 GPa, a failure strength of 70 MPa, and a failure strain of 5%.^47^ Further, the transparency allows one to see the cracks and inner layers of the assemblies.

Conventional rods as well as two‐ and three‐layered struts were tested. The two‐layer struts are assembled at core‐to‐shell ratios of 0.5 (*d*_1_ = 4 mm, *d*_2_ = 8 mm) and 0.71 (*d*_1_ = 5 mm, *d*_2_ = 7 mm). The three‐layer struts have a ratio of 0.5 and 0.7 with respect to the outer diameter (*d*_1_ = 5 mm, *d*_2_ = 7 mm, *d*_3_ = 10 mm). As all pairs of core diameter and inner shell diameter are identical for the layered struts, the gap distance is approximately zero. Three‐point bending experiments are conducted on a Zwick/Roell Z005 universal testing machine equipped with a 5 kN load cell at a test speed of 20 mm min^−1^. The test‐rig is equipped with revolving rollers of diameter 6 mm and set to a span length of *L* = 60 mm.

## Conflict of Interest

The authors declare no conflict of interest.

## Supporting information

Supplementary

Supplementary
