# Supplementary material for: Stiffness‐Independent Toughening of Beams through Coaxial Interfaces
Source: Adv Sci (Weinh). 2018 Oct 7;5(11):1800728. doi: 10.1002/advs.201800728 (PMC6247059; doi:10.1002/advs.201800728)
Supplement: Supplementary file 1 — Supplementary [file ADVS-5-1800728-s002.pdf]

Copyright WILEY-VCH Verlag GmbH & Co. KGaA, 69469 Weinheim, Germany, 2018.

Supporting Information

for *Adv. Sci.,* DOI: 10.1002/advs.201800728

Stiffness-Independent Toughening of Beams through Coaxial Interfaces

Jochen Mueller, Jordan R. Raney, Dennis M. Kochmann, and Kristina Shea*

Copyright WILEY-VCH Verlag GmbH & Co. KGaA, 69469 Weinheim, Germany, 2016.

Supporting Information

Stiffness-independent Toughening of Beams through Coaxial Interfaces

Jochen Mueller, Jordan R. Raney, Dennis M. Kochmann, Kristina Shea*

Figure S1 shows the bending tests of the specimens with outer diameters 8 mm (a) and 7 mm (b), as taken from the load-displacement curves shown in Figure 2b and Figure 2c. The behavior is similar to the one seen for the CSS strut in Figure 2a: upon the emergence of the first crack, the shell fails completely and the core continues to bear load until its own fracture strain is reached.

In addition to the analytical model, the experimentally tested specimens are simulated numerically using a commercially available, implicit FEM solver. Two symmetries (about the vertical axis in the beam cross-section and about the beam midpoint) are added to reduce the computational time. The bottom nodes in contact with the bottom fixture are fixed in all degrees of freedom, and the top nodes of the top fixture in *X* and *Y*, while the latter are displaced vertically downward according to the imposed deflection. The dimensions are identical to the ones shown in Figure 2a, and the mesh size is set to 0.2 mm with C3D8R linear brick elements with reduced integration. The friction between the specimens and the steel fixtures is set to 0 as roll fixtures are used in the experimental set-up, and the friction coefficient between the different layers of the PMMA specimens is set to 0.5. The simulations are stopped before the emergence of cracks and analyzed visually (Figure S2) and quantitatively in terms of their load-displacement data (Figure S3).

Figure S2 reveals that the stresses are comparable to the ones expected from the material data and those of the analytical results. The addition of layers does not significantly affect the stress distribution, which confirms our hypothesis. Further, there is no obvious shear observed in any of the cross-sections, which supports the use of Euler beam theory in our model. The analysis of the load-displacement curves shows similar moduli for all pairs of the same outer diameter (Figure S3). In addition, the curves overlap and only minor deviations are seen with increasing deflection.

For a linear-elastic material model, the optimal core-to-shell ratio to yield maximum energy absorption can be derived analytically. Within a linear-elastic regime, the energy absorption is calculated as

$$U = \frac{1}{2} D (F_{core} + F_{shell}),$$

(1)

where D is the deflection and F_{core} and F_{shell} the respective loads of the core and shell. The deflection can be expressed as a function of the ultimate strain, ε_u, as

$$D = \frac{\varepsilon_u L^2}{6 d_{core|shell}},$$

(2)

where L the span length and $d_{core|shell}$ the diameter of the core or shell, respectively. With the Euler-Bernoulli beam theory, the load of the core is calculated as

$$F_{core|shell} = \frac{8 \sigma_u I_{core|shell} L}{d_{core|shell}}$$

(3)

where σ_u is the ultimate strength of the material and $I_{core|shell}$ the second moment of area of the core or shell, respectively. Combining Equations 1, 2, and 3 yields

$$U_1 = a \left(d_{core}^3 + \left(d_{shell}^3 - \frac{d_{core}^4}{d_{shell}} \right) \right)$$

(4)

where

$$a = \frac{\sigma_u \pi \epsilon_u L}{96 * d_{shell}}$$

(5)

is constant under the assumption of $d_{shell} = const.$ The (local) maxima are calculated via the derivatives of Equation 4. The core-to-shell ratio of the maximum energy absorption is found to be $\frac{d_{core}}{d_{shell}} = 0.75$ for all linear-elastic materials. In a generic expression, the analytical formula to calculate the energy absorption for n gaps is

$$U_n = a\left(d_{gap\ 1}^3 + \sum_1^n \left(d_{gap\ n+1}^3 - \frac{d_{gap\ n}^4}{d_{gap\ n+1}}\right)\right),$$

(6)

where gap is the index of gaps $1 \dots n$. In the example of one gap from above, $n = 1$, hence $d_{gap\ 1} = d_{core}$ and $d_{gap\ n+1} = d_{shell}$. Solving Equation 6 for two variables yields 0.62 and 0.83 as optimal core-to-shell ratios for the first and second gap, respectively.

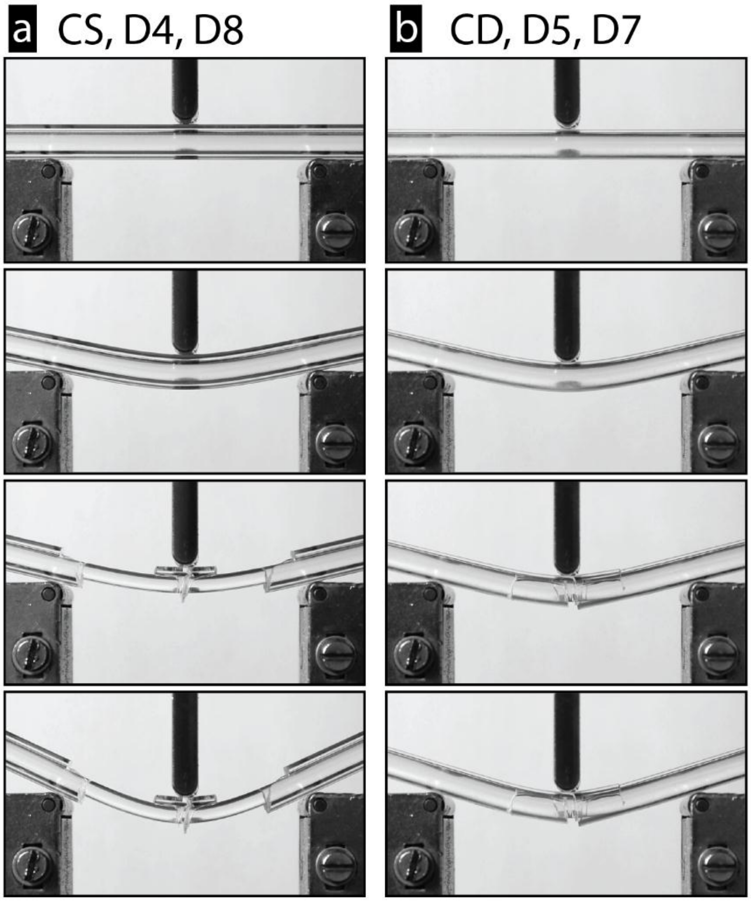

Figure S1. Flexure tests of struts with outer diameters 7 mm and 8 mm. Exemplary failures for outer diameters 8 mm (a) and 7 mm (b) are shown, which correspond to the load-displacement curves shown in Figure 2c and Figure 2c, respectively. The behavior is similar to the one of the CSS strut in Figure 2a: upon occurrence of the first crack, the shell fails completely and the core is capable of continuing to bear load until its own fracture strain is reached.

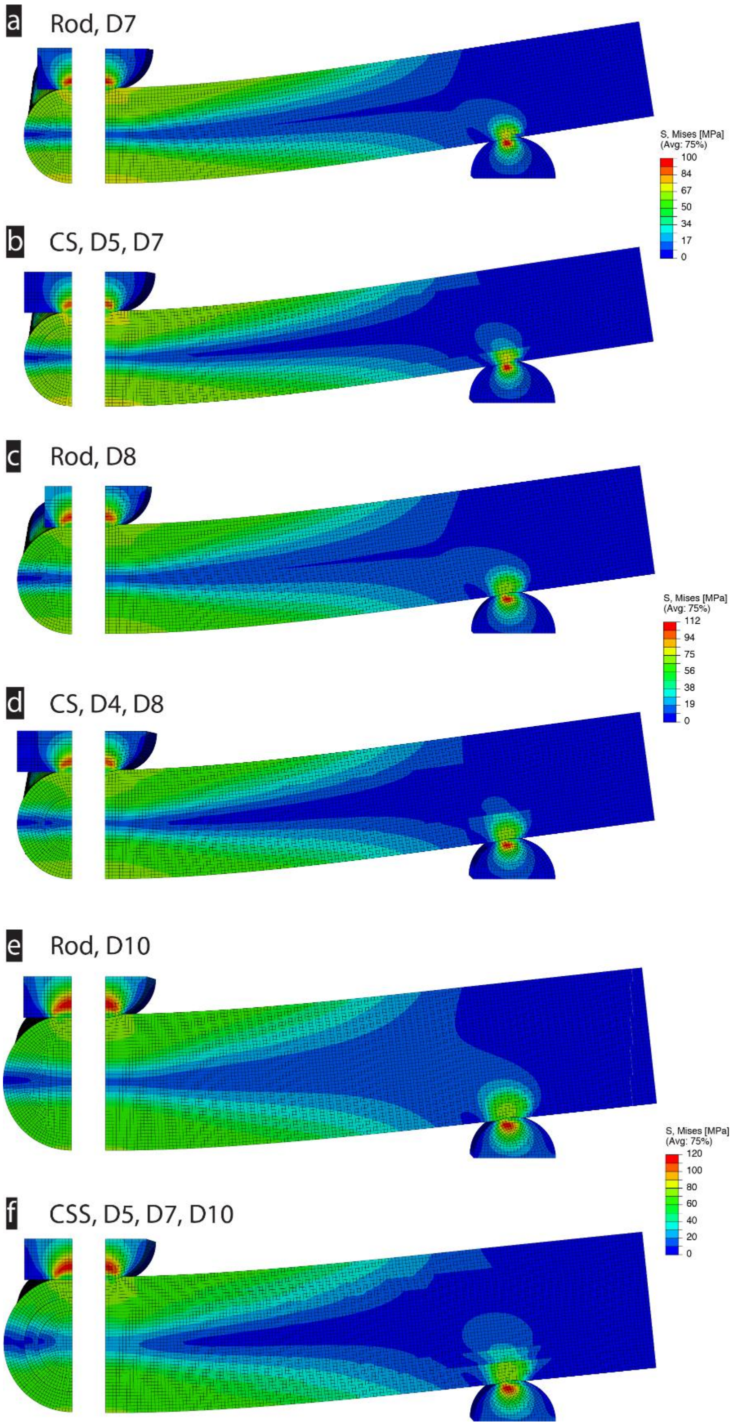

Figure S2. FEM analysis. Cross-sectional cuts of the specimens are shown for the rod-core-shell pair of outer diameters 7 mm (a, b), 8 mm (c, d), and 10 mm (e, f). For the core-shell (CS) and core-shell-shell (CSS) architectures, the intersection thickness is set to zero. In all subfigures, the left pane shows the transverse plane and the right pane the longitudinal plane. The shown stresses are similar

to the expected stresses from the analytical model and from the material data, and the addition of layers hardly affects the shown von Mises stress distribution.

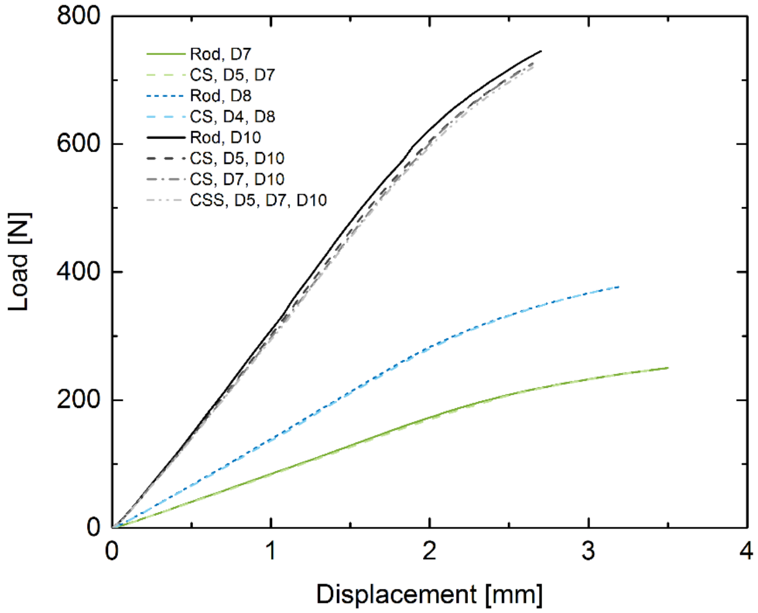

Figure S3. Load-displacement graphs of the FEM analysis. Load-displacement curves for the experimentally tested specimens are shown for the outer diameters of 7 mm (green), 8 mm (blue), and 10 mm (grey). The simulations are terminated before the emergence of cracks. For all pairs, the modulus is approximately identical and only minor deviations are seen with increasing displacement.

Movie S1. Conventional beams versus core-shell beams. The left hand side of the video shows a conventional beam of outer diameter 12 mm in three point bending. On the right side, a core-shell beam with three layers is shown, which fails in a stepwise manner, significantly adding to the toughness.
